# GRECC Connect Increases Access to Geriatric Specialty Care for Rural, Older Veterans With Complex Care Needs

**DOI:** 10.1093/geroni/igaa057.2884

**Published:** 2020-12-16

**Authors:** Kathryn Nearing, Stuti Dang, Eileen Dryden, Laura Kernan, Lauren Moo, Camilla Pimentel

**Affiliations:** 1 University of Colorado Anschutz Medical Campus, Aurora, Colorado, United States; 2 Miami VA Healthcare System, Miami, Florida, United States; 3 Center for Health Care Implementation Research (CHOIR), Bedford, Maryland, United States; 4 Center for Health Care Implementation Research (CHOIR), Newburyport, Maryland, United States; 5 Bedford VA Medical Center, Bedford, Massachusetts, United States; 6 Edith Nourse Rogers Memorial Veterans Hospital, Center for Heallthcare Organization and Implementation research, Bedford, Massachusetts, United States

## Abstract

A higher percentage of Veterans in rural areas are older, have multiple chronic conditions and select the VA for healthcare. To address the needs of rural older Veterans with complex needs, GRECC Connect hubs use case finding approaches combined with regular outreach and education to VA community-based outpatient clinic (CBOC) providers serving rural Veterans and caregivers. Alignment of GRECC Connect services with needs of providers and patients promotes establishment of therapeutic alliances in caring for medically complex older Veterans. After identifying high risk, high need patients, hubs use the following strategies to increase access to geriatric specialty care through telehealth modalities: 1) Co-management of patients through e-consultation and telehuddles (GRECC Connect interprofessional geriatric specialty care teams extend support to CBOC providers); 2) Clinical video telehealth to CBOCs and Video on Demand to Veteran homes (to reduce travel burden); and, 3) Tele-group visits (especially for behavioral health and caregiver support).

